# Effects of a novel ANLN E841K mutation associated with SRNS on podocytes and its mechanism

**DOI:** 10.1186/s12964-023-01218-w

**Published:** 2023-11-13

**Authors:** Li Lin, Yuhong Ye, Haidong Fu, Weizhong Gu, Manli Zhao, Jingmiao Sun, Zhongkai Cao, Guoping Huang, Yi Xie, Fei Liu, Lu Li, Qiuyu Li, Jianhua Mao, Lidan Hu

**Affiliations:** 1 Department of Nephrology, The Children’s Hospital, Zhejiang University School of Medicine, National Clinical Research Center for Child Health, 3333 Binsheng Road, Binjiang District, Hangzhou, 310003 Zhejiang China; 2 Department of Pathology, The Children’s Hospital, Zhejiang University School of Medicine, 3333 Binsheng Road, Binjiang District, Hangzhou, Zhejiang 310003 China

**Keywords:** ANLN mutation, SRNS, Podocyte, Podocyte-specific knockout mouse

## Abstract

**Background:**

Steroid-resistant nephrotic syndrome (SRNS) is characterized by unrelieved proteinuria after an initial 4–8 weeks of glucocorticoid therapy. Genes in podocytes play an important role in causing SRNS.

**Objective:**

This study aimed to report a pathogenic mutation in SRNS patients and investigate its effects on podocytes, as well as the pathogenic mechanism.

**Methods:**

We screened out a novel mutation by using whole-exon sequencing in the SRNS cohort and verified it via Sanger sequencing. Conservative analysis and bioinformatic analysis were used to predict the pathogenicity of the mutation. In vitro, stable podocyte cell lines were constructed to detect the effect of the mutation on the function of the podocyte. Moreover, an in vivo mouse model of podocyte *ANLN* gene knockout (*ANLN*^podKO^) was used to confirm clinical manifestations. Transcriptome analysis was performed to identify differential gene expression and related signaling pathways.

**Results:**

ANLN E841K was screened from three unrelated families. ANLN E841K occurred in the functional domain and was predicted to be harmful. The pathological type of A-II-1 renal biopsy was minimal change disease, and the expression of ANLN was decreased. Cells in the mutation group showed disordered cytoskeleton, faster cell migration, decreased adhesion, increased endocytosis, slower proliferation, increased apoptosis, and weakened interaction with CD2 association protein. *ANLN*^podKO^ mice exhibited more obvious proteinuria, more severe mesangial proliferation, glomerular atrophy, foot process fusion, and increased tissue apoptosis levels than *ANLN*^flox/flox^ mice after tail vein injection of adriamycin. Upregulated differentially expressed genes in cells of the mutation group were mainly enriched in the PI3K-AKT pathway.

**Conclusion:**

The novel mutation known as ANLN E841K affected the function of the ANLN protein by activating the PI3K/AKT/mTOR/apoptosis pathway, thus resulting in structural and functional changes in podocytes. Our study indicated that ANLN played a vital role in maintaining the normal function of podocytes.

Video Abstract

**Supplementary Information:**

The online version contains supplementary material available at 10.1186/s12964-023-01218-w.

## Introduction

Nephrotic syndrome (NS) is a group of diseases characterized by massive proteinuria, hypoalbuminemia, edema, and hyperlipidemia [1]. According to statistics, the global incidence of idiopathic nephrotic syndrome in children is approximately 10–50 per 100,000. NS is classified as either steroid-sensitive nephrotic syndrome or steroid-resistant nephrotic syndrome (SRNS) based on the responses to sterol treatment, among which the incidence of SRNS accounts for 2.1–27.3% [2, 3]. SRNS patients still demonstrate positive urinary protein after an initial 4–8 weeks of glucocorticoid therapy. Approximately 36–50% of SRNS patients will progress to end-stage renal disease (ESRD) within 10 years [4, 5]. Therefore, the diagnosis and management of SRNS have attracted the attention of pediatric nephrologists for a long period of time and have been represented a research hotspot worldwide.

Podocyte injury is a key factor in SRNS. Podocytes are involved in building the glomerular filtration membrane mechanical barrier and charge barrier [6]. Additionally, the podocytes play an important role in maintaining glomerular basement membrane (GBM) metabolic balance, opening capillary loops, hydrostatic pressure, and the synthesis of the GBM matrix. Podocyte-related gene mutations often lead to podocyte lesions, accompanied by diffuse thinning and stratified tearing of the GBM [7, 8]. Podocyte injury leads to abnormal glomerular function and structure [9]. Although the pathogenesis of SRNS is not fully understood, more than 70 podocyte-related genes are associated with SRNS [10, 11], including podocyte slit diaphragm (SD) proteins, transcription factor proteins, nuclear porins, cytoskeletal structure and related pathway proteins, mitochondrial-associated proteins, lysosomal proteins, and basement membrane-associated proteins. To determine the causes and pathogenesis of clinical SRNS in children and improve diagnosis and treatment, we collected blood samples from SRNS patients for whole-exon sequencing (WES) and discovered a novel mutation (ANLN E841K) related to three probands from different families.

Anillin (ANLN), which is encoded by the *ANLN* gene, was discovered as an F-actin binding protein in Drosophila in 1995 [12]. *ANLN* is localized on chromosome 7, and encodes a 124-kD highly conserved scaffold protein with multidomain proteins [13]. As reported, ANLN R431C significantly changed the function and structure of podocytes [14, 15]. This study aimed to clarify the effects of the ANLN E841K mutation on podocytes and its pathogenic mechanism for the potential treatment strategy and prognosis evaluation of SRNS children with this mutation.

## Methods

### Case enrollment

All families were accessed at the Children’s Hospital of Zhejiang University School of Medicine, Hangzhou, China. The institutional review boards of the Children’s Hospital of Zhejiang University School of Medicine (2020-IRB-057) approved this study. Informed and written consent was obtained from all of the human participants in this study. Moreover, the blood and urine of patients were collected. Patients who were diagnosed as SRNS presented the following characteristics. Patients had continuous proteinuria with ≥ 50 mg/kg per 24 h or urinary protein/creatinine ratio ≥ 2.0 mg/mg with hypoalbuminemia (25 g/L). Additionally, they still had proteinuria after 4–8 weeks of treatment with the maximum dose of prednisone. Patients who were over 18 years of age or who had been diagnosed with Alport syndrome were excluded. Kidney biopsy tissue was obtained, after which the tissue structure was observed by using hematoxylin–eosin (HE) staining and transmission electron microscopy (TEM). The protein expression of ANLN was observed via tissue immunofluorescence staining.

### Whole exome sequencing and sanger sequencing

Peripheral blood was collected, and genomic DNA was extracted by using the Blood Genome Column Medium Extraction Kit (Kangweishiji, China) according to the kit instructions. The extracted DNA samples were subjected to quality control by using a Qubit 2.0 fluorimeter and electrophoresis with a 0.8% agarose gel for DNA sample inspection. Liquid hybridization of the genomic DNA was performed by using Roche Nimble Gen Seq EZ Exome Enrichment Kit V2.0 and Seq EZ Exome Enrichment Kit V2.0 capture probes (Roche, USA). Moreover, the target DNA fragments were enriched to construct an exome library covering 19,119 genes with whole exons and flanking introns. Each enriched region shared 40 Mb of targeted sequences. High-throughput sequencing was performed by using an Illumina NovaSeq 6000 series sequencer (PE150), and no less than 99% of target regions were sequenced. The sequencing process was performed by the Zhiyin Oriental Translational Medicine Research Center. Raw data were cleaned after adapters removing, low-quality reads filtering and other quality control protocol. The clean data were aligned to the NCBI human reference genome (hg19) using BWA and variants were called using GATK. Samtools and Pindel were used to call SNPs (Single Nucleotide Polymorphisms) and indels, respectively. The clean data were than filtered, according to the quality of the sequencing, for further protocol. Nonsynonymous substitutions and SNPs with Minor Allele Frequency lower than 5% were filtered using Sorting Intolerant From Tolerant (SIFT). The function of mutated genes and their pathogenicity were then analyzed referencing to Database of the Single Nucleotide Polymorphism Database, 1000 Genomes Project, the Exome Aggregation Consortium, NHLBI Exome Sequencing Project, Online Mendelian Inheritance in Man, Swiss-var, Human Gene Mutation Database (HGMD), ClinVar and other disease databases. Furthermore, potential disease-causing variants identified by WES were confirmed by Sanger sequencing. Exon primer sequences are listed in Table S1. All of the sequences were analyzed with DNASTAR software.

### Damaging effect prediction and conservative analysis of mutation

The E841K variant in the ANLN gene was entered into online mutation analysis (Protein Variation Effect Analyzer, Polymorphism Phenotyping V-2, MutationTaster, and SIFT) to examine the predicted damaging effect of the amino acid substitution on the function of ANLN. The ANLN protein sequences of 11 species were downloaded from the UniProt database. The protein sequences of all of the species required for comparison were input to the T-coffee website and submitted to obtain the degree of variation of the ANLN E841K mutation among the different species.

### Human podocyte culture and establishment of stable overexpression cell lines

We obtained the conditionally immortalized human podocyte cell line from Otwo Biotech. Cells were cultured at 33 °C in RPMI 1640 medium supplemented with 10% fetal bovine serum and 10 U/mL mouse recombinant IFN-γ (R&D, USA) to propagate the podocytes. Podocytes were grown under nonpermissive conditions at 37 °C in the absence of IFN-γ for 14 days to induce differentiation. The open reading frame of Flag-WT (*ANLN*, NM_018685) was purchased from Youbio Co., Ltd. Flag-E841K was constructed via homologous recombination and primers are listed in Table S2. Additionally, Sanger sequencing validated the induced mutation. Given the low transient transfection efficiency of podocytes, we used homologous recombination to insert PCR products (*ANLN*_*WT*_ or *ANLN*_*E841K*_ gene fragment) into the vector of the pLJM1 plasmid. The utilized primers are listed in Table S3. The constructed plasmids and the vector pLJM1 plasmid were coated with lentivirus by using a 3-plasmid system. The pLJM1-EGFP, pLJM1-*ANLN*_WT_, and pLJM1-*ANLN*_E841K_ plasmids were packaged into lentiviruses and infected into podocytes at an MOI of 40 according to the instructions, wherein they were named Vector, WT, and E841K, respectively. Fresh medium was added 24 h after infection. Afterwards, complete medium containing 2.5 µg/mL puromycin was added for 72 h. Western blot (WB) and RT-qPCR were used to verify whether the target gene could be successfully expressed. Podocyte stable overexpression cell lines were used in all of the cell experiments (except for the pull-down experiment).

### Immunofluorescence

Cells seeded on 12-well coverslips (Solarbio, China) were washed with PBS in triplicate, fixed with 4% paraformaldehyde (PFA) at room temperature (RT) for 15 min, washed with PBS in triplicate, and blocked with 5% BSA in 1% Triton for 1 h. The cells were incubated with the primary antibody for 2 h at RT, washed with PBS three times, incubated with the secondary antibody for 1 h at RT, washed with PBS, dyed with DAPI for 5 min, washed with PBS, and then sealed. Moreover, the cells were maintained away from light during the procedure. Observations were made under a fluorescence microscope (Olympus, Japan).

### Wound healing assay

Cells at 37 ℃ were seeded in 6-well plates and cultured to 100% confluence. Cell monolayers were washed, and scratch wounds were applied by using a 1 mL pipet tip. Subsequently, the cells were cultured with serum-free medium and immediately imaged by using a Nikon microscope at 0, 12, 24, and 36 h at the same location after wound creation. ImageJ software was used to calculate the wound area.

### Endocytosis

Cells at 37 ℃ seeded on coverslips were incubated with 50 µg/mL Alexa 647-dextran (Invitrogen, USA) for 1 h. Cells were washed with PBS three times to remove cell surface Alexa 647-dextran. Additionally, the coverslips were fixed with 4% PFA at RT for 15 min, washed with PBS, dyed with DAPI for 5 min, washed with PBS, and then sealed. Observations were made under a fluorescence microscope (Olympus, Japan). Furthermore, ImageJ software was used to calculate the intensity of the fluorescence.

### Adhesion

Cells at 37 ℃ were seeded in 6-well plates at 4 × 10^5^/mL and incubated for 1 h. The medium was removed, the cells were washed 3 times with PBS, fixed with 4% PFA for 10 min, and washed with PBS. Afterwards, the cells were observed under a microscope with a 10 × objective, and 20 fields were randomly observed and photographed. ImageJ software was used to count the number of adherent cells.

### Immunoprecipitation assay

HEK293T cells were grown in DMEM with 10% FBS in 60 mm dishes until 85% confluency, and plasmids (Flag Vector, Flag-WT, or Flag-E841K) were transfected into HEK293T cells by using liposomal transfection reagent (Yeasen, China) according to the kit instructions. Cells were harvested at 48 h and lysed. Subsequently, the lysate was collected and centrifuged at 14, 000 rpm for 30 min at 4 °C. 50 μL washed protein A/G incubated with CD2 association protein (CD2AP) overnight was added to the supernatant and rotated overhead at 4 °C overnight. Afterwards, the beads were centrifuged at 3,000 rpm for 1 min at 4 °C and washed with RIPA buffer five times. Proteins were eluted by boiling the beads in 2 × Laemmli sample buffer and separated via SDS‒PAGE.

### Cell cycle

Cells at 33 ℃ were collected by using centrifugation at 300 × g for 5 min and washed with cold PBS once at 4 °C. Cell pellets were then resuspended in 500 μL DNA staining solution and 5 μL permeabilization, after which they were incubated for 30 min at RT. After incubation, the cells were analyzed via flow cytometry (Beckman, USA) within 1 h.

### Cell proliferation

Cells at 33 °C were seeded onto 96-well plates at 5,000/well and further incubated with complete medium supplemented with 10% CCK-8 solution for 0, 24, 48, 72, 96, and 120 h at 33 °C. Following incubation, the cell viability was measured at the indicated time point according to the instructions. The optical density at 450 nm was detected by using a Multiskan FC (Thermo, USA).

### Apoptosis

Cells at 37 °C were seeded onto 6-well plates and cultured with serum-free medium for 24 h when they reached 90% confluence. Cells were detached by using trypsin-no EDTA and washed with cold PBS. Moreover, cell pellets for apoptosis were collected via centrifugation at 300 × g for 5 min, washed with cold PBS twice at 4 °C, resuspended in 100 μL 1 × binding buffer, and incubated with 5 μL 647-conjugated Annexin V and 10 μL PI (Annexin V-Alexa Fluor 647/PI Apoptosis Detection Kit) for 15 min at 37 °C. After incubation, cell pellets were resuspended in 400 µl of PBS. Cell analysis was performed on a flow cytometry (Beckman, USA) within 1 h.

### Western blot analysis

Cells or tissues were carefully washed with cold PBS. For lysis, RIPA buffer with cocktail (MCE, 1:100), PMSF (1:100), and DNAse I (1:1000) was added to the cells. The lysate was incubated for 30 min on ice and centrifuged at 14,000 rpm for 30 min at 4 °C. The cell lysates were then boiled for 5 min at 98 °C, and 20 μg of protein per sample was separated via SDS‒PAGE by using 10–12% gels. Afterwards, proteins were transferred to a PVDF membrane (Merck, Germany) and incubated in blocking buffer (5% skim milk powder dissolved in TBS containing 0.1% Tween 20 [TBST]) for 1 h at RT. Primary antibodies (Table S4) were diluted in TBST with 5% BSA and incubated at 4 °C overnight. Subsequently, the membrane was washed three times with TBST and incubated with diluted horseradish peroxidase–coupled secondary antibodies for 1 h at RT. After three washes with TBST, the signal was detected by using a Chemidoc Touch Imaging System (Bio-Rad, USA).

### RT‒qPCR

Total RNA was extracted from three different stable cell lines by using an Eastep® Super Total RNA Extraction kit (Promega, China). Total RNA was reverse transcribed to cDNA by using the Eastep® RT Master Mix kit (Promega, China). Quantitative real-time PCR was performed by using qPCR SYBR Green Master mix (Yeasen, USA). Moreover, gene amplification was performed by using a CFX96 Real-Time PCR System (Bio-Rad, USA) with the following reaction conditions: 1 cycle, 95 °C for 30 s; and 40 cycles, 95 °C for 10 s, and 60 °C for 30 s. A threshold cycle number for the gene of interest was calculated based on the amplification curve representing a plot of the fluorescence signal intensity versus the cycle number. The delta threshold cycle number was calculated as the difference between the threshold cycle number of the genes of interest between different cell lines. Furthermore, each value was normalized by the difference in the threshold cycle number for the housekeeping gene (Actin) amplification in the same samples. Primer sequences are included in Table S5.

### Mice and genotyping

All of the experimental procedures were approved by the Animal Care and Utilization Committee of Zhejiang University School of Medicine. Mice were raised in a pathogen-free environment with a 12 h light/dark cycle and allowed access to food and water ad libitum. Conditional ANLN knockout (*ANLN*^podKO^) mice were constructed by using CRISPR‒Cas9. To excise the floxed ANLN genes in the podocytes of the kidney, we used the Nphs2-Cre lines. For DNA isolation and genotyping, tissue was incubated in 150 μL of 50 mm NaOH at 98 °C for 30 min, and 50 μL of 0.5 M Tris–HCl (pH = 8.0) was added. The mixtures were centrifuged for 5 min, and the DNA-containing supernatant was used for genotyping with the primers ANLN mouse-F2, ANLN mouse-R2, ANLN mouse-F1, ANLN mouse-R1, NPHS2Cre mouse-F, and NPHS2Cre mouse-R to identify *ANLN*^podKO^ mice (Table S6). In addition, mouse tail DNA was amplified with ANLN Mouse-F2 and ANLN Mouse-R2 primers with only a 215 bp band and NPHS2Cre mouse-F and NPHS2Cre mouse-R primers with a 204 bp band. There was a 347 bp band of DNA in kidney tissue amplified with ANLN mouse-F2 and ANLN mouse-R1 PCR, thus indicating that *ANLN*^podKO^ mice were successfully constructed. Moreover, there were five mice in each group for the experiments. Twenty-four hours of urine from mice was loaded onto SDS gels to determine the proteinuria phenotype. The protein-creatinine ratio (μg/mg) was also measured.

### Tissue preparation and histopathologic analyses

Kidneys were perfused with PBS and immediately removed. One kidney was directly fixed with 2.5% glutaraldehyde for the TEM analysis. Part of the other kidney was fixed with 4% PFA overnight at RT for paraffin sectioning. Sections were cut to 2 μm thickness and stained with hematoxylin–eosin (HE), periodic acid-Schiff (PAS), and Masson’s trichrome (MASSON). Moreover, glomerular injury was assessed by counting the total number of glomeruli with segmental injury. Part of the other kidney was immediately frozen for sectioning. Sections were cut to 10 μm thickness, and the expression of ANLN was detected by using immunofluorescence. Slit diaphragm injury was determined by counting the number of slit diaphragms per micrometer of GBM in TEM images of at least 200 μm GBM per animal. Furthermore, adriamycin (ADR)-induced kidney injury was established in control and conditional knockout mice by injecting a single dose of ADR (20 mg/kg) via the tail vein. Urine and serum samples were collected prior to sacrifice, and kidneys were removed for further analysis. Part of the renal cortex tissue was stored in liquid nitrogen for WB and RT‒qPCR.

### RNA sequencing analysis

Total RNA was extracted from WT and E841K stable cell lines by using an Eastep® Super Total RNA Extraction kit (Promega, China). RNA integrity and concentration were verified by using an Agilent 2100 Bioanalyzer (Agilent, USA). The mRNA was isolated via the NEBnext Poly (A) mRNA Magnetic Isolation Module (NEB, USA). Additionally, the cDNA library was constructed following the manufacturer’s instructions of the NEBNext Ultra RNA Library Prep Kit for Illumina (NEB, USA) and NEBNext Multiplex Oligos for Illumina (NEB, USA). In brief, the enriched mRNA was fragmented into approximately 200 nt RNA inserts, which were used to synthesize the first-strand cDNA and the second cDNA. The double-stranded cDNA was subjected to end-repair/dA-tail and adaptor ligation. Moreover, suitable fragments were isolated by using Agencourt AMPure XP beads (Beckman, USA) and enriched via PCR amplification. Finally, the constructed cDNA libraries were sequenced on a flow cell by using an Illumina HiSeq™ sequencing platform. The clean reads that were filtered from the raw reads were mapped to the honeybee (Apis mellifera) genome (OGSv3.2) by using TopHat2 (Kim, Pertea et al. 2013) software. Furthermore, the aligned records from the aligners in BAM/SAM format were further examined to remove potential duplicate molecules. Gene expression levels were estimated by using FPKM values (fragments per kilobase of exon per million fragments mapped) via Cufflinks software (Trapnell, Williams et al. 2010). Deseq (Anders and Huber 2010) and Q-values were employed to evaluate differential gene expression between queen and worker honeybees. Afterwards, gene abundance differences between those samples were calculated based on the ratio of the FPKM values. The false discovery rate (FDR) control method was used to identify the threshold of the P value in multiple tests to compute the significance of the differences. Herein, only genes with an absolute value of log2fold change (FC) ≥ 0.5 and FDR significance score ≤ 0.05 were used for the subsequent analysis. To annotate the gene with gene ontology (GO) terms, the Nr BLAST results were imported into the Blast2 GO program (Conesa, Gotz et al. 2005). This analysis mapped all of the annotated genes to GO terms in the database and counted the number of genes associated with each term. The obtained annotation was enriched and refined by using topgo (R package). Furthermore, Kyoto Encyclopedia of Genes and Genomes (KEGG) pathways were assigned to the assembled sequences via the Perl script.

### Statistical analysis

The statistical analyses were performed with GraphPad Prism 6. All of the data are presented as the means ± standard errors. One-way ANOVA was used to determine differences between groups. *P* < 0.05 was considered to be statistically significant.

## Results

### ANLN E841K was identified as a disease-causing mutation

**Fig. 1:**
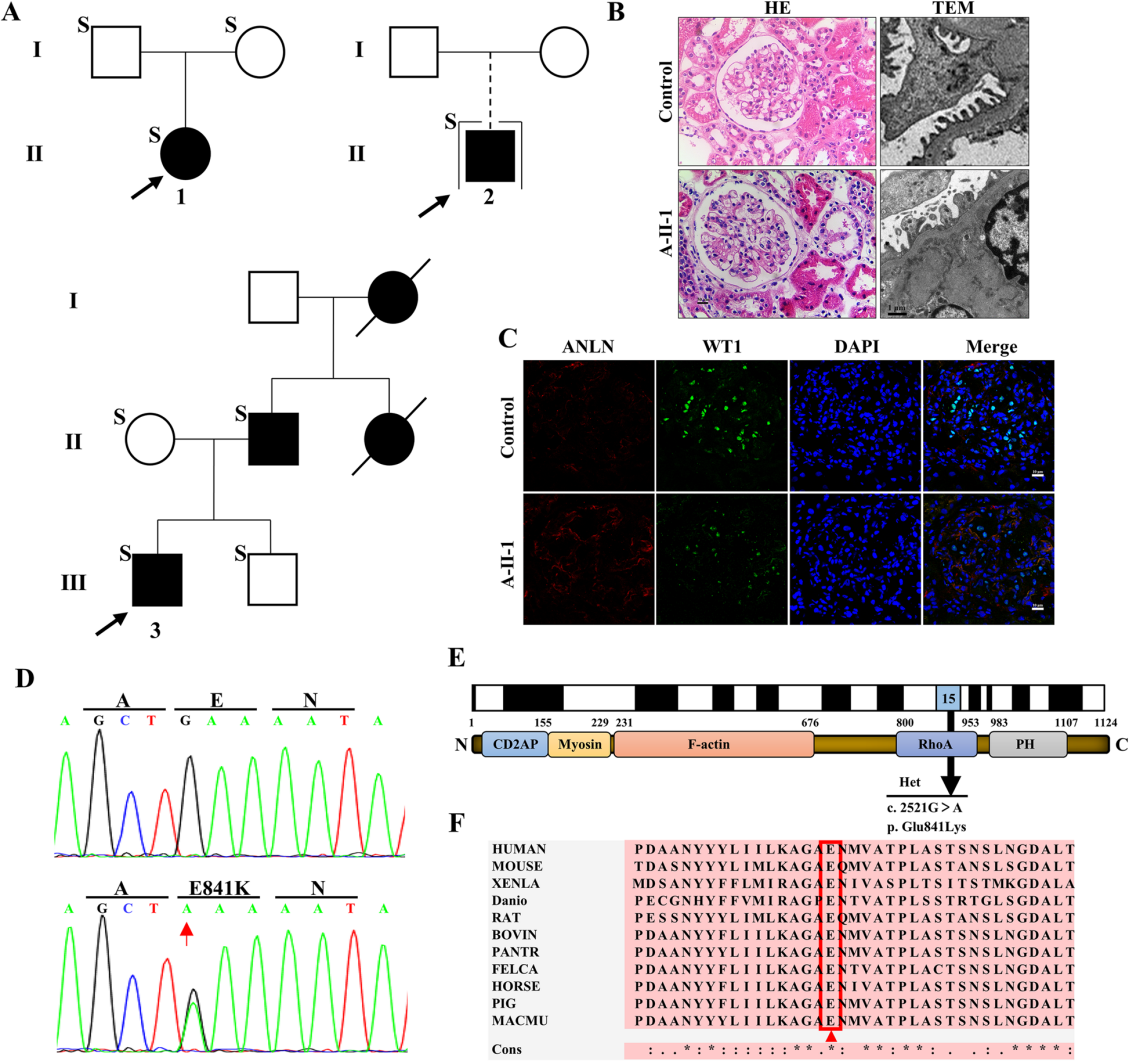
Identification of ANLN c.2521 G > A mutation in pedigrees with SRNS. **a** Three probands related to SRNS were screened, and DNA samples were available from individuals labeled S, which were analyzed by WES. The black arrow indicates proband. ●: female patient. ■: male patient. **b** Representative HE and TEM images of kidney biopsy histology from proband 1 showed an area of minimal-change disease. The scale bar was 50 μm for HE and 1 μm for TEM. **c** Missense heterozygous mutation ANLN c.2521 G > A (p. E841K) occurred in exon 15. Top, mutant sequence; bottom, wildtype sequence. **d** Representative immunofluorescence staining of Control and proband A-II-1 kidney tissue. The co-localization of ANLN and WT1 in the kidney tissue of the proband A-II-1 was similar to that of the negative control without ANLN primary antibody incubation. Red represents ANLN, green WT1, and blue DAPI. The scale bar was 50 μm. **e** The mutated nucleotide is located in the 15th exon and mutated amino acid is located in the RhoA domain indicated by the black arrow. **f** The E841 residue is conserved in evolution between different species. The mutated residue is labeled with red triangles. Conserved residues (*); highly similar residues (:); similar residues (.)

According to the WES results of children diagnosed with SRNS in the Department of Nephrology of our hospital, we screened out the de novo mutation involved in three unrelated children with the same mutation site, which was identified as *ANLN* c.2521 G > A (p. Glu841Lys). Clinical findings in affected individuals (Table 1) show that ANLN_E841K_ is involved in proteinuria that cannot be relieved by glucocorticoids alone. There were four affected individuals in the family of proband 3 (Fig. 1a). Both male and female individuals were consistently affected with an autosomal dominant inheritance pattern (Fig. 1a). Additionally, Fig. 1b is a representative sample of kidney biopsy from proband 1. This result indicated that individual glomeruli were mildly dilated, and foot processes were effaced. As one of the podocyte-specific marker proteins, WT1 is located in the podocyte nucleus, and ANLN is also mainly located in the cell nucleus. Therefore, we can evaluate the expression of ANLN in podocytes via the colocalized expression of ANLN and WT1 in the nucleus. We used the sections (normal tissue adjacent to renal Wilms' tumor) that were not incubated with ANLN primary antibody and incubated with the corresponding fluorescent secondary antibody as the control. Compared with the control group (normal tissue adjacent to renal Wilms' tumor), the colocalization of ANLN and WT1 was rarely observed in the biopsy kidney tissue of proband A-II-1 (Fig. 1c). Additionally, the DNA from three probands and the parents of probands 2 and 3 were demonstrated to have the same amino acid mutation in *ANLN* and confirmed by using Sanger sequencing (Fig. 1d). A nonsynonymous heterozygous mutation *ANLN* c.2521 G > A (p. E841K) occurred in exon 15 (Fig. 1e). Moreover, the E841 residue was conserved in evolution among various species (Fig. 1f). With the gene base at 2521 changing from G to A, the 841 amino acid changed from glutamic acid (acidic) to lysine (basic), which may have effects on the structure and function of the protein. The population frequencies of ANLN E841K are 0.0096 in the Single-Nucleotide Polymorphism database, 0.0064 in 1000 Genomes, and 0.00067 in Exome Aggregation Consortium. The damaging score of ANLN E841K was -3.13 (deleterious) in the Protein Variation Effect Analyzer, 1.0 (likely damaging) in Polymorphism Phenotyping V-2, 1.0 (disease causing) in MutationTaster, and 0.015 (damaging) in Sorting Intolerant From Tolerant. There were 11 ANLN mutation cases in the HGMD so far and ANLN E841K had not been reported. The population frequencies and damaging score indicated that ANLN E841K was a disease-causing mutation.

**Table 1:** Clinical characteristic of three probands

ID	Gender	Age (y)	Proteinuria	Sensitive to steroid	Renal biopsy	Current treatment	Current status
1	Female	4	Yes	No	MCD	Prednisone and Tacrolimus	Stable control
2	Male	5	Yes	No	No	Unknown	Unknown
3	Male	8	Yes	No	FSGS	Prednisone and Tacrolimus	Unknown

### The overexpression of ANLN E841K led to podocyte dysfunction

**Fig. 2:**
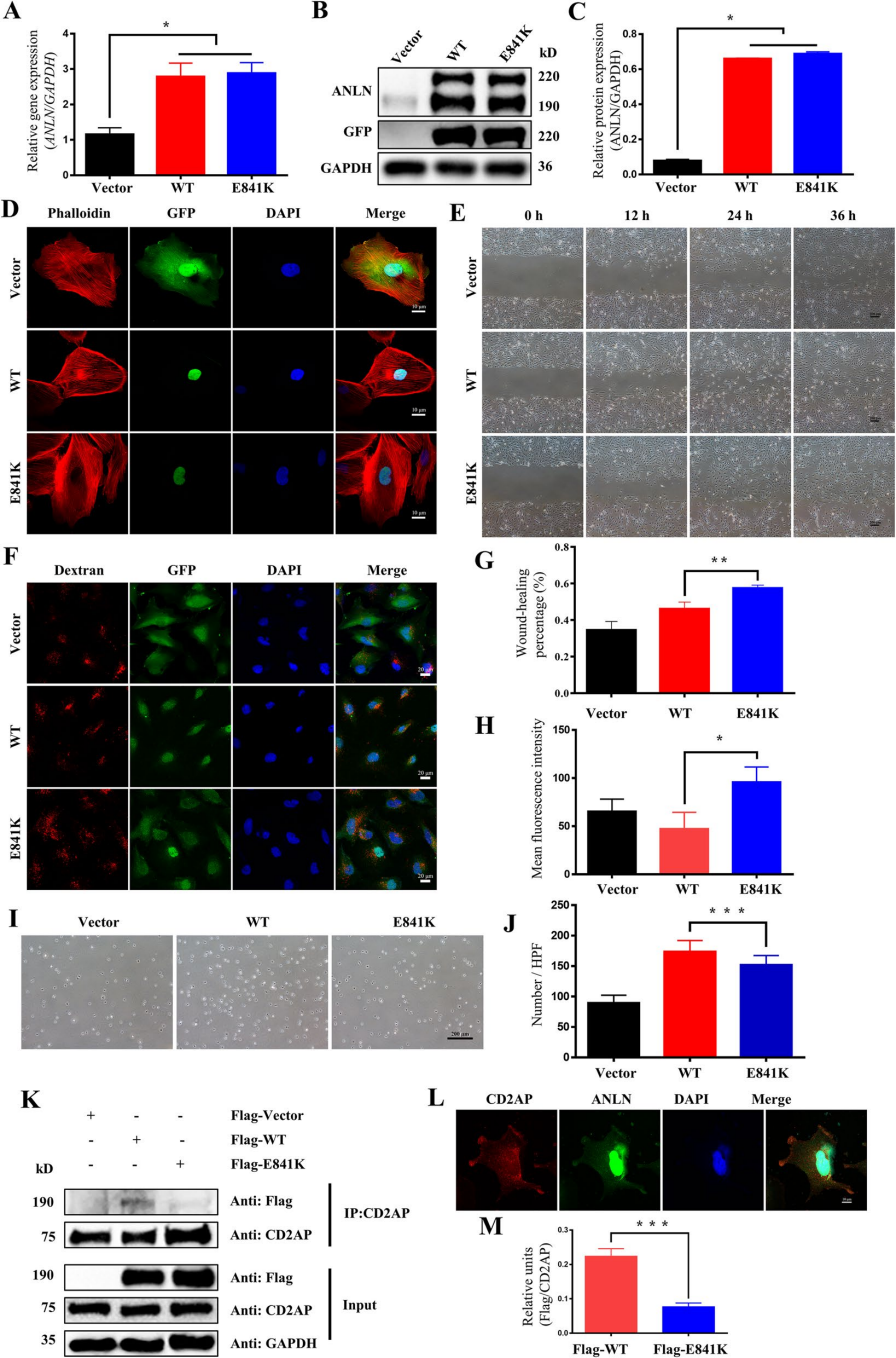
The effect of overexpressed ANLN E841K on the phenotype of podocytes. **a** Gene expression quantification of *ANLN* in three groups of cells were tested by RT‒qPCR. **b**-**c** Representative WB plots and WB quantification showed that podocyte gene overexpression cell lines were successfully constructed. **d** Representative immunofluorescence images of phalloidin. Red stands for Phalloidin, green for GFP, and blue for DAPI. The cytoskeleton of the mutant group was clustered and arranged disorderly. **e**, **g** Representative pictures of three group cells at the same position on plate at 0, 12, 24, and 36 h, respectively. The cell migration speed of E841K group was significantly faster than that of the WT group. **f**, **h** Representative immunofluorescence images of endocytosis. Red stands for Dextran, green for GFP, and blue for DAPI. The fluorescence intensity of podocytes with dextran in E841K was significantly higher than the other two groups. **i**, **j** Representative pictures of Vector, WT and E841K cells adhering to the 6-well plate. The adhesion ability of E841K cells was significantly reduced compared with that of the WT cells. **k-m** ANLN E841K interacted with CD2AP. **k** Representative WB plots of protein samples from the three groups of cells. **l** Representative fluorescence images of the three group cells. Red stands for CD2AP, green for ANLN, and blue for DAPI. **m** WB quantification of Flag protein pulled down by CD2AP. The amount of Flag protein pulled down by CD2AP in the mutant group was significantly less than that in the WT group. * indicates *P* < 0.05, ** indicates *P* < 0.01, and *** indicates* P* < 0.001

Podocyte cell lines were constructed to express pLJM1, pLJM1-*ANLN*_WT_, and pLJM1-*ANLN*_E841K_; in addition, they were screened with puromycin and were termed Vector, WT, and E841K, respectively. Proteins and RNAs were extracted from the three cell groups, and their relative expression levels were detected by using WB and RT‒qPCR. The results showed that the mRNA expression levels of WT and E841K were significantly higher than those of the vector (Fig. 2a), and there was one more band in the WB of the WT and E841K groups than in the vector group (Fig. 2b, c).

ANLN could bind to a variety of cytoskeletal proteins; therefore, we performed immunofluorescence staining with Phalloidin-iFluor 594 on the three groups of cell lines to observe the effects of mutation on the cytoskeleton. The results showed that the cytoskeleton in the mutant group was arranged in a disorderly manner (Fig. 2d), thus indicating that the mutation affected the binding of ANLN to cytoskeletal proteins. We subsequently performed a scratch assay to examine the motility of the three overexpressed cell lines. The results of the scratch assay showed that the E841K group had a higher reduction ratio of scratch area than the WT group at 12 and 24 h after scratching, thus indicating that the E841K group had a faster migration speed (Fig. 2e, g). These findings suggested that ANLN may play an important role in podocyte motility. To examine the effect of the mutation on the endocytic function of the cells, we incubated the overexpressing cell lines with medium containing the dextran probe. The results showed that the fluorescence intensity of podocytes in the E841K group was significantly higher than that in the Vector and WT groups, and the difference was statistically significant, thus indicating that the mutation resulted in increased endocytosis (Fig. 2f, h). Moreover, an adhesion assay was used to detect the adhesion ability of the three cell lines. The results showed that the number of adherent cells in the E841K group was less than that in the WT group, thus indicating that the E841K mutation could weaken the cell adhesion ability (Fig. 2i, j). Due to the fact that previous studies have demonstrated that CD2AP interacts with ANLN, immunofluorescence showed that CD2AP colocalized with ANLN (Fig. 2l), and pull-down assays showed that the binding of ANLN protein to CD2AP in the E841K group was less than that in the WT group (Fig. 2k, m).

**Fig. 3:**
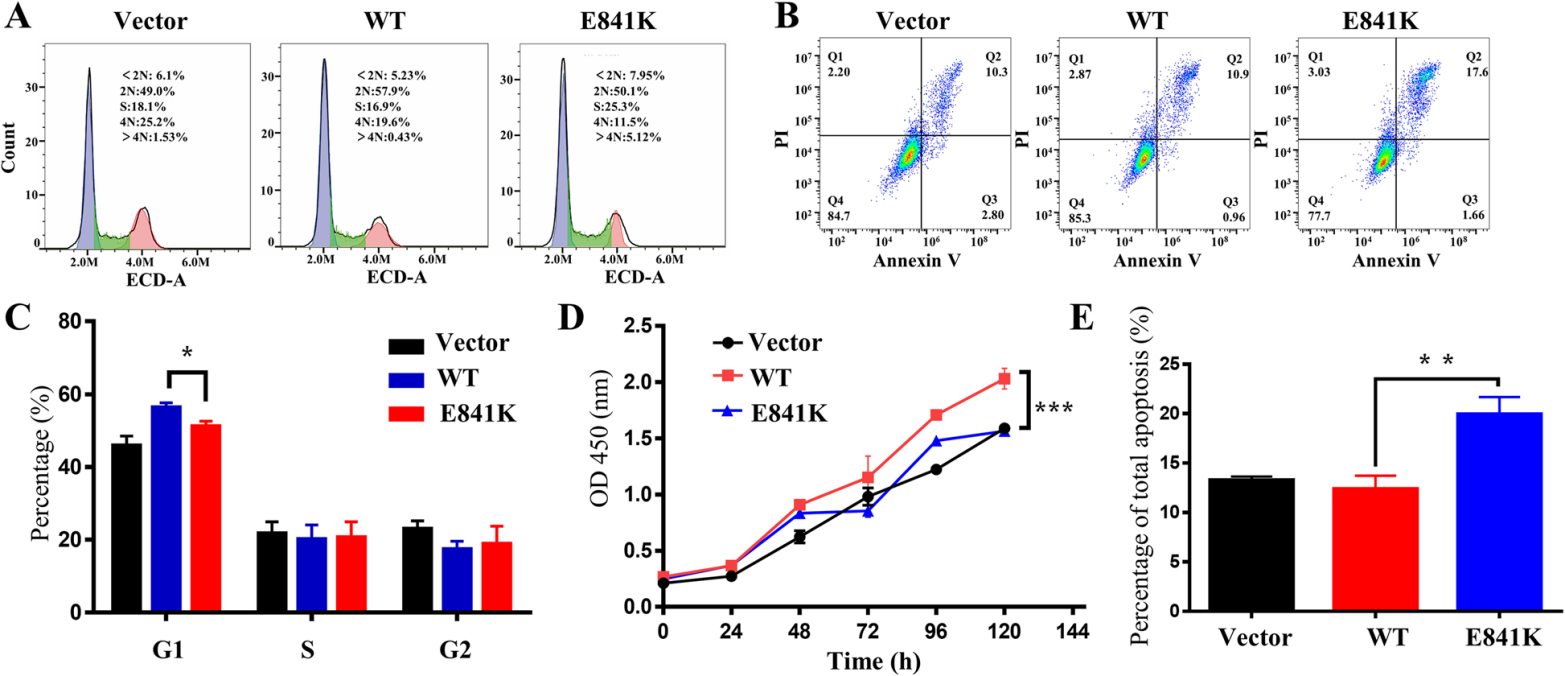
The effect of overexpressed ANLN E841K on function of podocytes. **a**, **c** Flow cytometry of cell cycle showed that the cells of E841K group were arrested in G2 phase. **d** The cell proliferation rate of the E841K group was significantly lower than that of the WT at 96 and 120 h. **b**, **e** Flow cytometry of apoptosis analysis revealed that percentage of total apoptosis of E841K group was significantly higher than that of the WT. * indicates *P* < 0.05, ** indicates *P* < 0.01, and *** indicates *P* < 0.001

ANLN is a protein that stabilizes shrinkage rings during cytokinesis. Therefore, flow cytometry was used to detect the cell cycle of the three podocyte-stable cell lines. The cells in the E841K group were less in the G1 phase (Fig. 3a, c) and expressed more p21, p27, and Cyclin E2 proteins (Fig. S1a-d). We further performed cell proliferation and apoptosis experiments, which are closely related to the cell cycle. The CCK-8 results demonstrated no significant difference between E841K and WT cells at 24 and 48 h but gradually exhibited differences at 96 and 120 h. Additionally, the proliferation rate of the E841K group was faster than that of the WT group (Fig. 3d). The proportion of total apoptosis in the E841K group was significantly higher than that in the vector and WT groups (Fig. 3b, e).

### *ANLN*^podKO^ mice showed more severe damage after ADR treatment

**Fig. 4:**
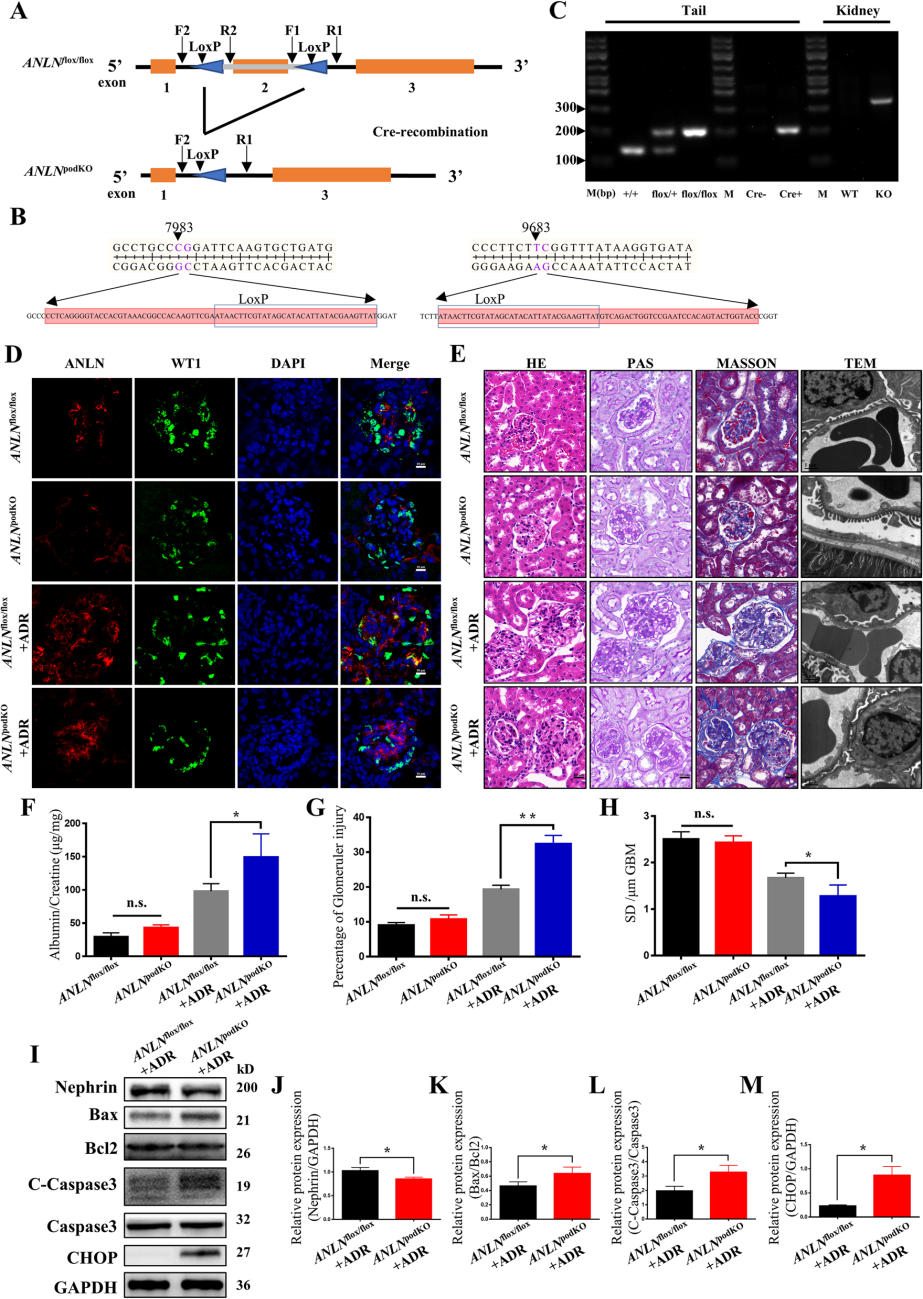
Kidney changes in glomerular *ANLN*^podKO^ mice. **a** Flow chart of the construction of *ANLN*^podKO^ mice. loxP indicates the site of gRNA insertion. F1, R1, F2, and R2 represent primer. **b** Sanger sequencing of the mouse ANLN gene inserted gRNA. **c** Representative agarose gel electrophoretogram of PCR products of mouse tail and kidney tissue DNA. M indicates Marker, + / + indicates no loxP site, flox/ + indicates that one allele is inserted with gRNA, flox/flox indicates that both alleles are inserted with gRNA, WT indicates that the fragment in the middle of the two loxP sites is not deleted, and KO indicates that the fragment in the middle of the two loxP sites is deleted. **d** Representative immunofluorescence images of glomerulus. Red stands for ANLN, green for WT1, and blue for DAPI. The scale bar was 10 μm. **e** Representative images of HE, PAS, Masson staining and TEM observation in four groups. The scale bar was 20 μm for HE and 1 μm for TEM. **f** The urine Albumin/Creatinine ratio of *ANLN*^flox/flox^, *ANLN*^podKO^, *ANLN*^flox/flox^ + ADR and *ANLN*^podKO^ + ADR mice. **g** Quantification of glomerular injury in four groups. **h** Quantification of slit diaphragm number on GBM in four groups. **i** Representative WB plots of kidney tissue in *ANLN*^flox/flox^ and *ANLN*^pod−KO^ mice after ADR treated. **j**-**m** WB quantification of Bax/Bcl2, C-Caspsase3/Caspsase3, CHOP in *ANLN*^flox/flox^ + ADR and *ANLN*.^podKO^ + ADR mice groups. n.s. indicates *P* > 0.05, * indicates *P* < 0.05, and ** indicates *P* < 0.01

To evaluate the role of *ANLN* in podocytes in vivo, we bred *ANLN*^podKO^ with transgenic mice that express Cre recombinase under the control of the NPHS2 promotor. All of the mice were maintained in a pathogen-free facility and received food and water ad libitum. Exon 2 of the mouse ANLN gene was knocked out, and subsequent exons could not be properly translated (Fig. 4a). The sequencing results are shown in Fig. 4b. The *ANLN*^podKO^ mice were identified via DNA PCR (Fig. 4c). In the *ANLN*^podKO^ group, no ANLN colocalized with WT1, thus indicating that *ANLN* was successfully knocked out in podocytes (Fig. 4d). During 36 weeks of continuous analyses of renal phenotype and glomerular structure, there was no difference in body weight and proteinuria between the *ANLN*^podKO^ and *ANLN*^flox/flox^ mice (Fig. S2a-b). It is worth noting that the *ANLN*^podKO^ mice exhibited more severe glomerular injury, foot process effacement, and fewer SDs (Fig. S2c-f). Afterwards, 20 mg/kg ADR was used to simulate stress conditions, and the results showed that it worked (Fig. S2g-i). After ADR treatment for 4 weeks, *ANLN*^podKO^ mice showed more severe proteinuria than *ANLN*^flox/flox^ mice (Fig. 4f, Fig. S2j). Moreover, kidney section staining of ADR-treated *ANLN*^podKO^ mice demonstrated more cellular infiltrates and glomerular injury than that of ADR-treated *ANLN*^flox/flox^ mice (Fig. 4e, g). Ultrastructural analyses via TEM elucidated that podocyte foot processes of ADR-treated *ANLN*^podKO^ mice were more seriously effaced and disordered (accompanied by a reduction in the SD number per GBM) than those of ADR-treated *ANLN*^flox/flox^ mice (Fig. 4e, h). Tissue WB indicated that the protein expression levels of nephrin and apoptosis markers, including Bax/Bcl2, C-Caspase3/Caspase3, and CHOP, were significantly increased compared with those in the control group (Fig. 4i-m).

### ANLN E841K affected cells through the PI3K/AKT pathway

**Fig. 5:**
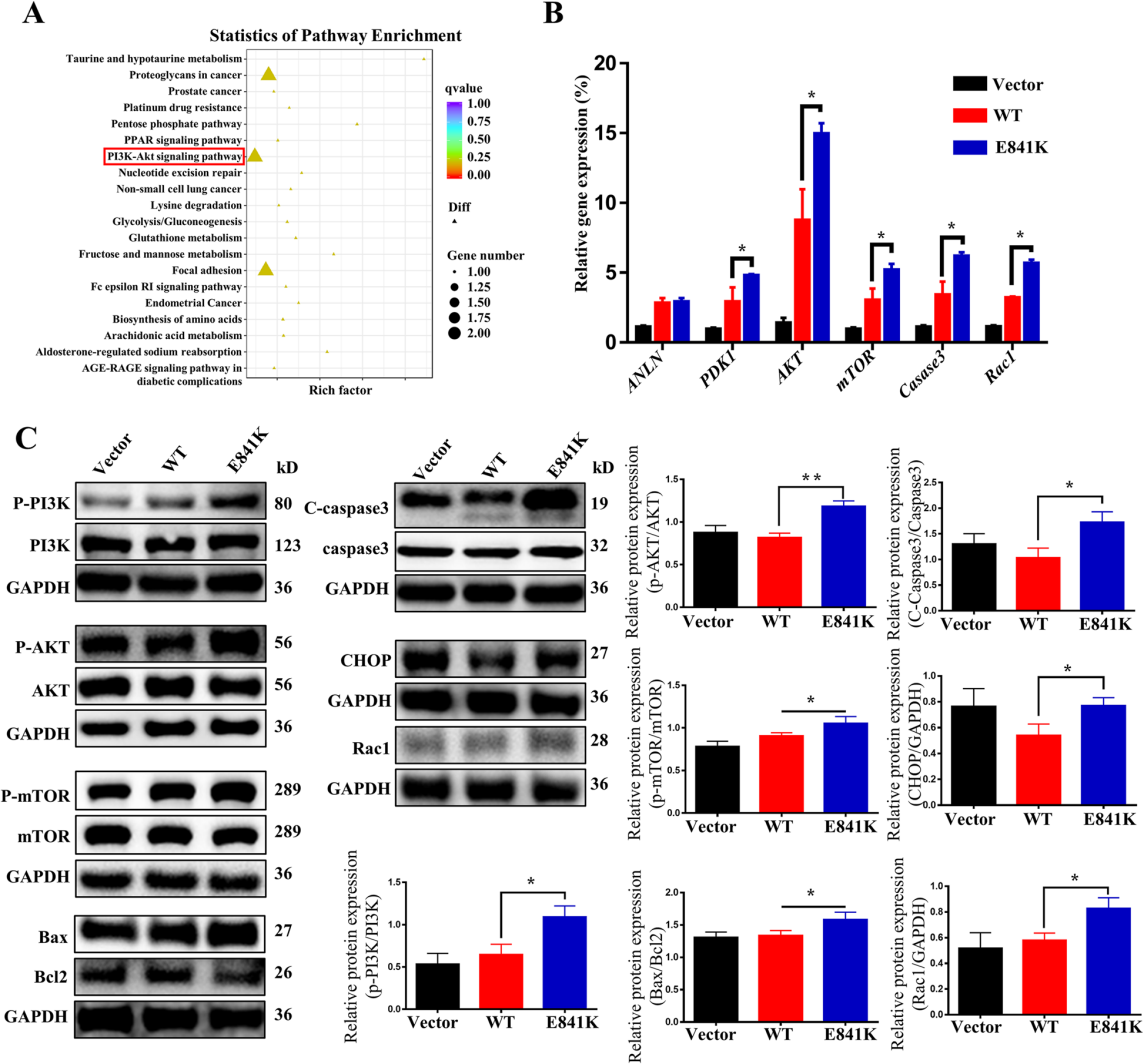
RNA seq and pathway verification. **a** Statistics of pathway enrichment between WT and E841K cell samples showed that up regulated differential expression gene enriched in PI3K/AKT pathway. **b** Relative gene expression of the cells of Vector, WT, and E841K groups. **c** Representative WB plots and quantification of the three group cells. * indicates *P* < 0.05 and ** indicates *P* < 0.01

The abovementioned results indicated that the E841K mutation significantly impacted the function of podocytes. Therefore, transcriptome sequencing was performed to determine how the mutation affects the cells. According to the results, a volcano map was made to directly show the differentially expressed genes found in the WT and E841K groups (Fig. S3). The screening criteria for differentially expressed genes were FDR ≤ 0.05 and log_2_FC ≥ 0.5. Moreover, a total of 1,029 differentially expressed genes were screened out. Additionally, a total of 613 genes were significantly upregulated, and 418 genes were significantly downregulated in E841K compared with WT. According to the KEGG annotation results, the top 20 pathways are listed in Fig. 5a.

To further verify the results, we extracted cell RNA and performed RT‒qPCR to detect the expression levels of *ANLN*, *PDK1*, *AKT*, *mTOR*, *Caspase-3*, and *Rac1*. As shown in Fig. 5b, the gene expression levels of *PDK1*, *AKT*, *mTOR*, *caspase-3*, and *Rac1* in the E841K group were upregulated compared with those in the WT group. Subsequently, we performed WB to detect protein expression and found that PI3K, AKT, mTOR, Bax/Bcl2, C-Caspase3/Caspase3, CHOP, and Rac1 were upregulated compared with those in the WT group (Fig. 5c). Generally, the loss-of-function ANLN E841K mutation may exert its deleterious effects on podocyte function and viability through multiple derangements of PI3K/AKT signaling.

## Discussion

SRNS refers to nephrotic syndrome that cannot be alleviated by the management of sufficient glucocorticoids for 4–8 weeks [3]. Currently, there is no clear explanation for the pathogenesis of SRNS in children. At present, an increasing number of studies have focused on podocyte-related genes causing SRNS, and *ANLN* is one of one of these genes [10]. Only 3 *ANLN* gene mutations associated with SRNS have been currently reported [14–16]. The onset age of the patients ranged from 9- to 69-years-old, and they presented different disease progression even in the same family. The age distribution of the uremia stage of these patients was between 35- and 75-years-old [14]. ANLN E841K in proband 3 was identified to be inherited from his father, who was normal until adulthood, which was consistent with those data reported above. In addition, proband 2 was effectively treated with hormone combined with tacrolimus; however, urine protein appeared again under stressful conditions, such as cold, fatigue, or diarrhea. These results further indicated that the new mutation ANLN E841K may be a susceptible variant to SRNS, and the carriers of this variant are required to demonstrate clinical symptoms under specific stress conditions. Moreover, ANLN may not be the only factor causing SRNS; however, it plays a role in the occurrence and development of SRNS, which is consistent with the characteristics of susceptible variants. Based on the abovementioned evidence, we conclude that ANLN variant NM 018685, c.2521 G > A (p. E841K), is a susceptible variant of SRNS.

Podocytes are terminally differentiated cells, and podocytes physiologically exit the cell cycle. As previously reported, the expression of ANLN in podocytes was significantly higher in FSGS patients and HIVAN nephropathy mice than in normal individuals and mice [14]. There is a dynamic balance between the synthesis and degradation of ANLN protein in podocytes. The balance may be broken under stressful conditions, and podocytes attempt to reenter the cell cycle for self-repair. We found that the cells of the E841K group were arrested in G2 phase, and the proliferation rate of the E841K group was slower, thus indicating that the ANLN E841K mutation could lead to abnormal cell division.

As a scaffolding protein that binds to a variety of cytoskeletal proteins, ANLN is involved in the functional activities of podocytes, such as cell adhesion, motility, and endocytosis by affecting cytoskeletal proteins [13, 17, 18]. In our study, we observed that the cytoskeleton of the mutant group aggregated to the plasma membrane and was unevenly arranged, thus indicating that the protein mutation would affect the arrangement of podocyte cytoskeleton proteins, thus resulting in the fusion and disappearance of foot processes and interfering with the filtering function of podocytes. Cells in the E841K group migrated faster and had a weaker adhesion ability than those in the WT group, thus indicating that the ANLN E841K mutation would more easily lead to the shedding of cells from the basement membrane and the formation of proteinuria. Related studies have shown that the ANLN protein domain can interact with CD2AP, which is an important podocyte structural protein [19, 20]. CD2AP is involved in cell adhesion and cell migration by interacting with various actin regulatory proteins, such as CAPZ, Rac1, and ANLN. In podocytes, CD2AP is a cohesive molecule that connects SD to cytoskeletal proteins and affects the migration function of cells [15]. In our study, the protein mutant binding to CD2AP became weaker compared with that of the WT group, and the migration speed of the E841K group was faster than that of the WT group. In our study, the mutation ANLN E841K is located in the RhoA domain of the protein. Rac1 is a member of the Rac subfamily of the Rho family. Moreover, Rac1 activation regulates cell phagocytosis, cell migration, vesicle transport, and other processes [21]. In this study, Rac1 activation was higher in the E841K group than in the WT group, which is consistent with the results of cell migration and endocytosis experiments. Podocyte injury can manifest as diminished or enhanced endocytic function. In addition, dynamic-deficient podocytes exhibit the accumulation of blocked endocytic pits [22], and the accumulation of blue-labeled albumin endocytic vesicles was observed in podocytes from a rat model of nephrotic syndrome induced by puromycin [23]. According to the cell phenotype, the mutation ANLN E841K can cause podocyte dysfunction.

Based on the results of cell experiments, we hypothesized that the mutation may cause loss of protein function. *ANLN*^podKO^ mice were generated to further explore the effects of ANLN on the structure and function of renal podocytes. In the ANLN knockdown experiment, the zebrafish showed severe edema and a large amount of protein loss. Moreover, podocyte foot process fusion or even disappearance could be observed under TEM [14]. In our study, no significant proteinuria was observed, and no obvious podocyte abnormalities were observed under TEM at 12 and 24 weeks in *ANLN*^podKO^ mice. In a zebrafish knockdown experiment, ANLN expression was decreased not only in glomerular podocytes, but also in glomerular endothelial cells, mesangial cells, and renal tubule cells, which seriously affected the filter membrane barrier and albumin reabsorption. Hypoalbuminemia and glomerular overload further aggravate podocyte injury. Moreover, ANLN plays an important role in stabilizing shrinkage rings during cytokinesis, and it is widely distributed throughout the body, especially in the nervous system [24, 25]. Patients with ANLN mutations do not show neurological symptoms either in reported cases or in our case. Given that the function of this gene is essential to maintain the normal life activities of cells and tissues, it may compensate for other similar genes [26]. We can conclude that the ANLN E841K mutation causes cellular dysfunction, but it can be incompletely compensated. However, more severe glomerular segmental sclerosis and foot process fusion were observed in the kidney tissue of 36-week-old *ANLN*^podKO^ mice. We believe that this may be because 36-week-old mice have entered the old age period; in this scenario, the body's various functions decline, and they are more likely to exhibit a disease state. It is worth noting that when *ANLN*^flox/flox^ and *ANLN*^podKO^ mice were treated with ADR, which represents a classic animal model of nephropathy [27], *ANLN*^podKO^ mice showed more obvious proteinuria and foot process fusion. The possible reason for this effect may be that inadequate compensation will be shown under stress conditions. All of these results indicate that ANLN plays a role in maintaining the function of podocytes.

Transcriptome sequencing results showed that the PI3K/AKT signaling pathway was upregulated. PI3K/AKT signaling is widely recognized as a prosurvival pathway. Interestingly, a significant increase in apoptosis percentage was observed in both E841K-overexpressing podocytes and in podocytes of *ANLN*^podKO^ mice treated with ADR. The PI3K/AKT pathway is involved in the regulation of a variety of important life activities of podocytes. CD2AP and nephrin can bind to the p85 regulatory subunit of PI3K, aggregate PI3K on the cytoplasmic membrane, and activate the PI3K-dependent AKT signaling pathway [28, 29]. Moreover, nephrin can induce the phosphorylation of several target proteins of AKT, including death promoter homolog (Bad) proteins of the Bcl-2 family. When phosphorylated Bad dissociated from Bcl-2 and bound to its binding site, it promoted cell survival at the mitochondrial level. It is known that CHOP is expressed at low levels under physiological conditions [30]. Previous studies have confirmed that the upregulation of mTOR leads to endoplasmic reticulum stress [31] along with the activation of the CHOP protein [32–35]. CHOP can induce a cascade of caspase molecules, and caspase-3 is a key molecule in the process of the final apoptosis of cells [36].

## Conclusion

In this study, we demonstrated that the loss-of-function ANLN E841K mutation may have a detrimental effect on podocyte function by upregulating the PI3K/AKT signaling pathway. Via ADR treatment of *ANLN*^podKO^ mice, we identified that ANLN played a role in maintaining the normal function of glomerular podocytes. This article clarified the pathogenicity of the ANLN E841K mutation and provided evidence for clinical diagnosis and treatment in SRNS related to the ANLN E841K mutation. Further studies will focus on potential effective therapies targeting molecules of the pathway.

### Supplementary Information

**Additional file 1:** **Fig. S1.** Representative WB plots and quantification of p21, p27, and Cyclin E2 in the three group cells. n.s. indicates *P* > 0.05, ** indicates *P* < 0.01, and *** indicates *P* < 0.001.

**Additional file 2: Fig. S2.** Kidney related phenotype of *ANLN*^podKO^ and ADR model mice. (a) Body weight analyze of *ANLN*^flox/flox^ and *ANLN*^podKO^ mice at 4, 8, 12, 24, and 36 weeks. (b) Urine representative SDS‒PAGE staining of *ANLN*^flox/flox^ and *ANLN*^podKO^ mice at 12, 24, and 36 weeks. (c, d) Representative HE images and glomerulosclerosis proportion of kidney tissues from 12, 24, and 36 weeks *ANLN*^flox/flox^ and *ANLN*^podKO^ mice. The scale bar was 20 μm. (e, f) Representative TEM images and SD number of kidney tissues from 12, 24, and 36 weeks *ANLN*^flox/flox^ and *ANLN*^podKO^ mice. The scale bar was 2 μm. (g) Urine representative SDS‒PAGE staining of Control and ADR treated mice. (h) BUN and Creatine of Control and ADR treated mice. (i) The tissue RT‒qPCR of TNF-α, IL-1β, and IL-6 in Control and ADR treated mice. (j) Urine representative SDS‒PAGE staining of *ANLN*^flox/flox^ and *ANLN*^podKO^ mice after treated with ADR. n.s. indicates *P* > 0.05, * indicates *P* < 0.05, and ** indicates *P* < 0.01.

**Additional file 3: Fig. S3.** Volcano plot of E841K verses WT cell samples. Red dots represent up-regulated genes and blue down-regulated genes.

**Additional file 4: Fig. S4.** Original and full-length blot images. (a) Uncropped blot images of ANLN, GFP, and GAPDH corresponding to Fig.3b. (b) Uncropped blot images of input including Flag, CD2AP, and GAPDH corresponding to Fig.3k. (c) Uncropped blot images of IP including Flag and CD2AP corresponding to Fig.3k. (d) Uncropped blot images of Nephrin, Bax, Bcl2, C-Caspase3, Caspase3, CHOP, and GAPDH corresponding to Fig.4i. (e) Uncropped blot images of P-PI3K, PI3K, and GAPDH corresponding to Fig.6c. (f) Uncropped blot images of P-AKT, AKT, and GAPDH corresponding to Fig.6c. (g) Uncropped blot images of P-mTOR, mTOR, and GAPDH corresponding to Fig.6c. (h) Uncropped blot images of Bax, Bcl2, and GAPDH corresponding to Fig.6c. (i) Uncropped blot images of C-Caspase3, Caspase3, and GAPDH corresponding to Fig.6c. (j) Uncropped blot images of CHOP and GAPDH corresponding to Fig.6c. (k) Uncropped blot images of Rac1 and GAPDH corresponding to Fig.6c. (l) Uncropped blot images of CyclinE2, P27, P21, and GAPDH corresponding to Fig.S1a.

**Additional file 5: Table S1.** Primer sequences for Sanger sequencing. **Table S2.** Primer sequences for plasmid construction. **Table S3.** Primer sequences for plasmid construction. **Table S4.** Dilution of antibody. **Table S5.** Primer sequences for RT-qPCR. **Table S6.** Primer sequences for Genotyping.

## Data Availability

All of the data that were generated or analyzed during this study are included in this published article.

## Abbreviations

NS: Nephrotic syndrome
SRNS: Steroid-resistant nephrotic syndrome
ESRD: End-stage renal disease
GBM: Glomerular basement membrane
SD: Slit diaphragm
WES: Whole-exon sequencing
WB: Western blot
CD2AP: CD2 association protein
HE: Hematoxylin–eosin
PAS: Periodic acid-schiff
MASSON: Masson’s trichrome
TEM: Transmission electron microscope
ADR: Adriamycin
FDR: False discovery rate
FC: Fold change
GO: Gene ontology
KEGG: Kyoto encyclopedia of genes and genomes
SNPs: Single Nucleotide Polymorphisms
HGMD: Human Gene Mutation Database
