# Supplementary figures and images for: Effects of a novel ANLN E841K mutation associated with SRNS on podocytes and its mechanism
Source: Cell Commun Signal. 2023 Nov 13;21:324. doi: 10.1186/s12964-023-01218-w (PMC10644598; doi:10.1186/s12964-023-01218-w)

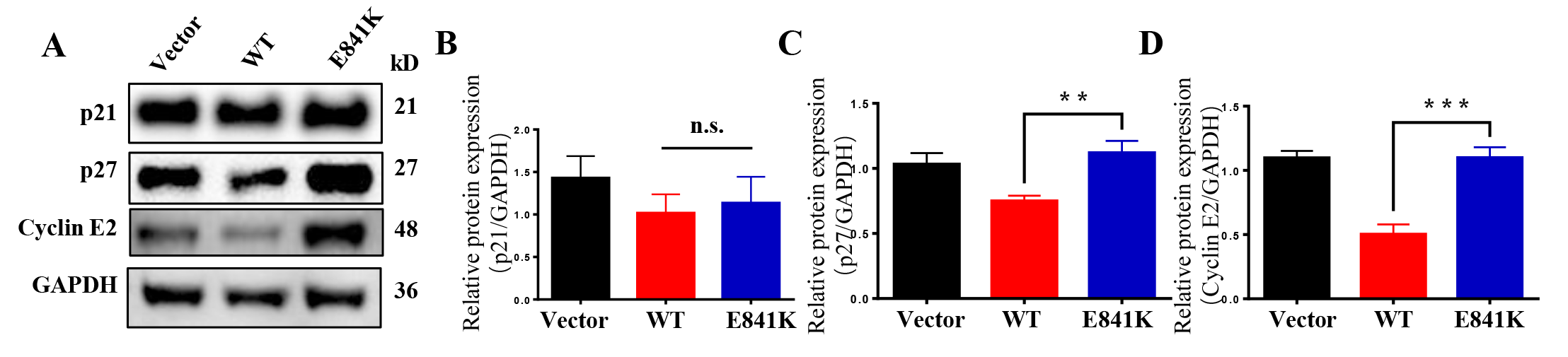

Supplement: Supplementary file 2 — Additional file 1: Fig. S1. Representative WB plots and quantification of p21, p27, and Cyclin E2 in the three group cells. n.s. indicates P > 0.05, ** indicates P < 0.01, and *** indicates P < 0.001. [file 12964_2023_1218_MOESM1_ESM.tif]

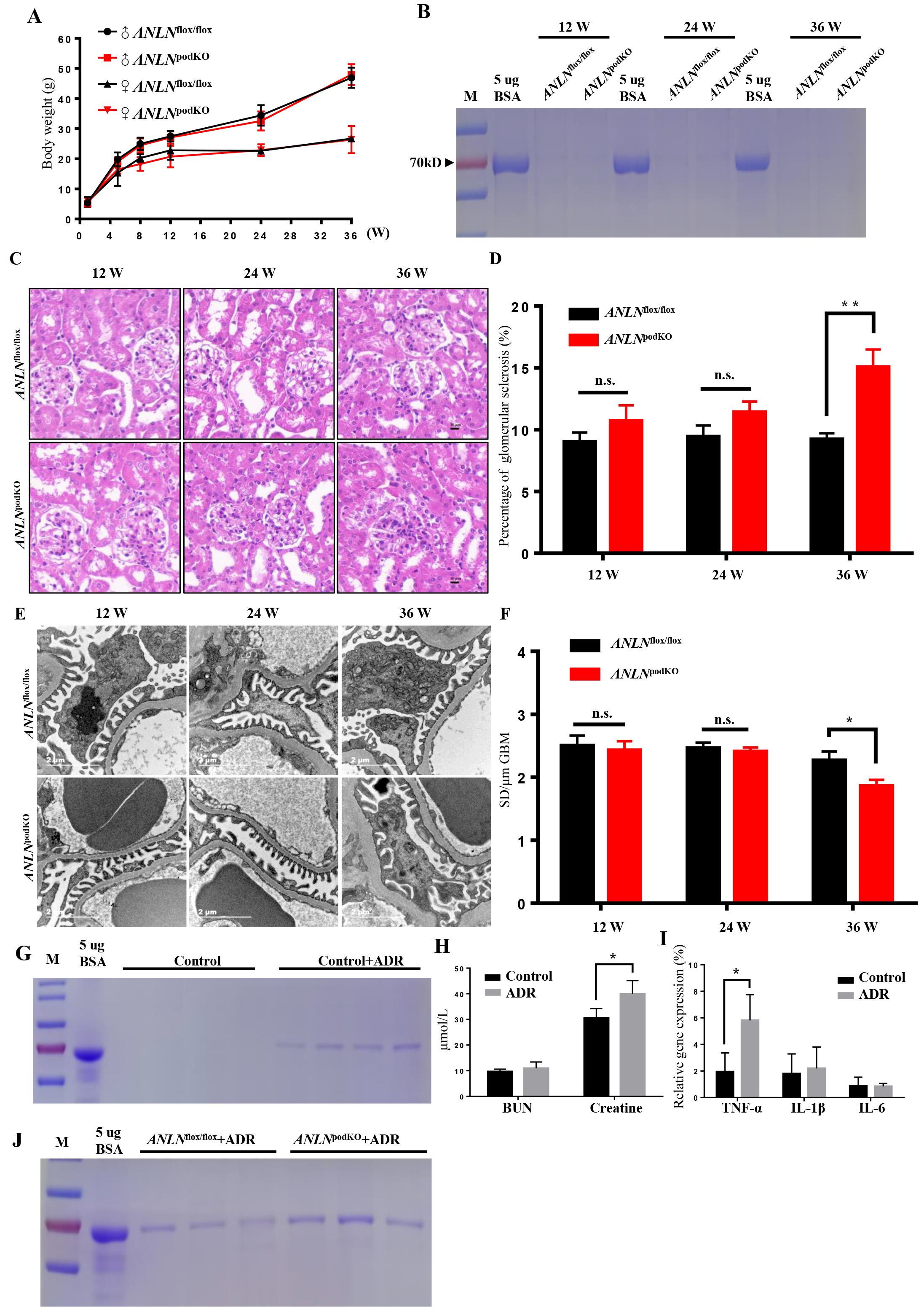

Supplement: Supplementary file 3 — Additional file 2: Fig. S2. Kidney related phenotype of ANLNpodKO and ADR model mice. (a) Body weight analyze of ANLNflox/flox and ANLNpodKO mice at 4, 8, 12, 24, and 36 weeks. (b) Urine representative SDS‒PAGE staining of ANLNflox/flox and ANLNpodKO mice at 12, 24, and 36 weeks. (c, d) Representative HE images and glomerulosclerosis proportion of kidney tissues from 12, 24, and 36 weeks ANLNflox/flox and ANLNpodKO mice. The scale bar was 20 μm. (e, f) Representative TEM images and SD number of kidney tissues from 12, 24, and 36 weeks ANLNflox/flox and ANLNpodKO mice. The scale bar was 2 μm. (g) Urine representative SDS‒PAGE staining of Control and ADR treated mice. (h) BUN and Creatine of Control and ADR treated mice. (i) The tissue RT‒qPCR of TNF-α, IL-1β, and IL-6 in Control and ADR treated mice. (j) Urine representative SDS‒PAGE staining of ANLNflox/flox and ANLNpodKO mice after treated with ADR. n.s. indicates P > 0.05, * indicates P < 0.05, and ** indicates P < 0.01. [file 12964_2023_1218_MOESM2_ESM.tif]

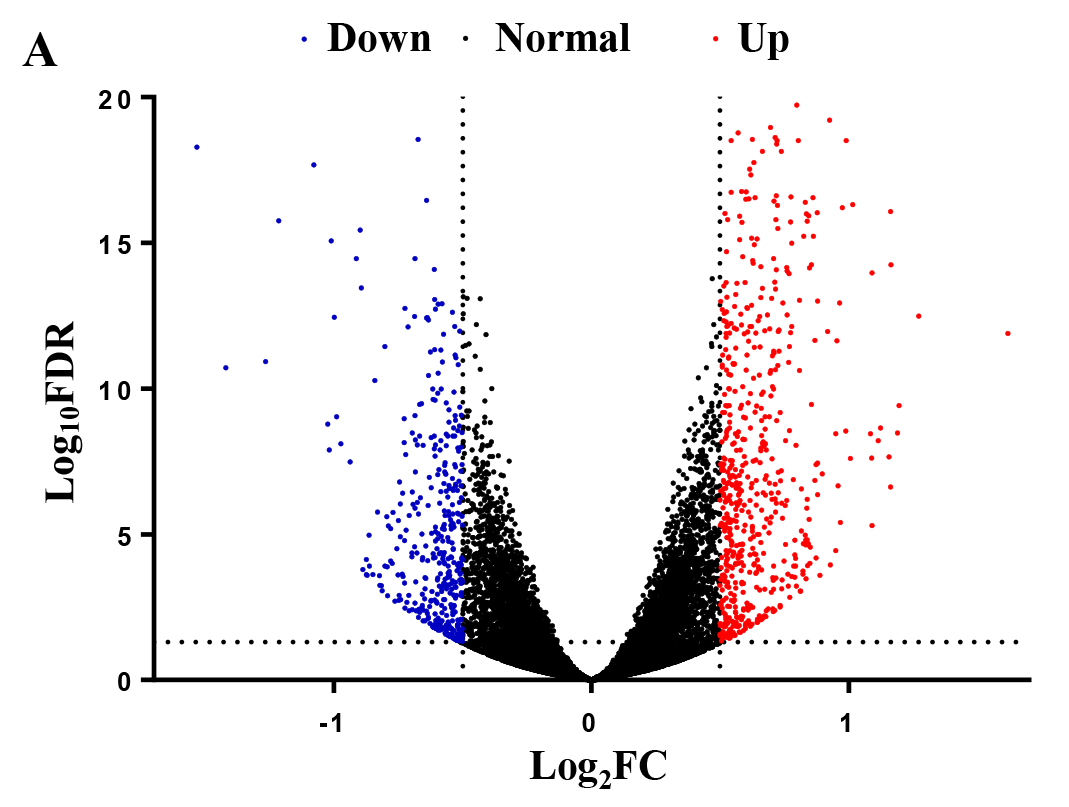

Supplement: Supplementary file 4 — Additional file 3: Fig. S3. Volcano plot of E841K verses WT cell samples. Red dots represent up-regulated genes and blue down-regulated genes. [file 12964_2023_1218_MOESM3_ESM.tif]

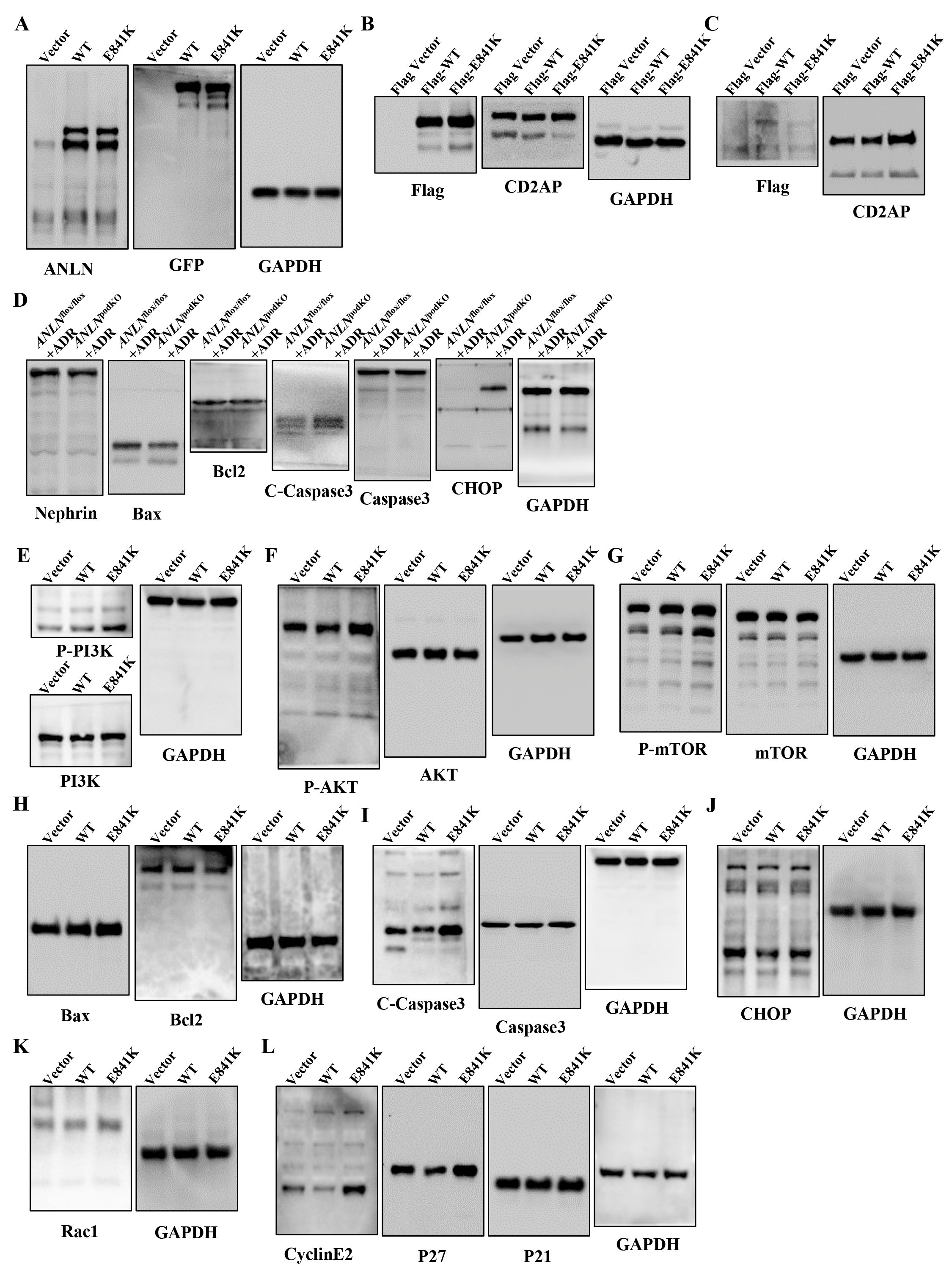

Supplement: Supplementary file 5 — Additional file 4: Fig. S4. Original and full-length blot images. (a) Uncropped blot images of ANLN, GFP, and GAPDH corresponding to Fig.3b. (b) Uncropped blot images of input including Flag, CD2AP, and GAPDH corresponding to Fig.3k. (c) Uncropped blot images of IP including Flag and CD2AP corresponding to Fig.3k. (d) Uncropped blot images of Nephrin, Bax, Bcl2, C-Caspase3, Caspase3, CHOP, and GAPDH corresponding to Fig.4i. (e) Uncropped blot images of P-PI3K, PI3K, and GAPDH corresponding to Fig.6c. (f) Uncropped blot images of P-AKT, AKT, and GAPDH corresponding to Fig.6c. (g) Uncropped blot images of P-mTOR, mTOR, and GAPDH corresponding to Fig.6c. (h) Uncropped blot images of Bax, Bcl2, and GAPDH corresponding to Fig.6c. (i) Uncropped blot images of C-Caspase3, Caspase3, and GAPDH corresponding to Fig.6c. (j) Uncropped blot images of CHOP and GAPDH corresponding to Fig.6c. (k) Uncropped blot images of Rac1 and GAPDH corresponding to Fig.6c. (l) Uncropped blot images of CyclinE2, P27, P21, and GAPDH corresponding to Fig.S1a. [file 12964_2023_1218_MOESM4_ESM.tif]
