# Supplementary material for: Effects of a novel ANLN E841K mutation associated with SRNS on podocytes and its mechanism
Source: Cell Commun Signal. 2023 Nov 13;21:324. doi: 10.1186/s12964-023-01218-w (PMC10644598; doi:10.1186/s12964-023-01218-w)
Supplement: Supplementary file 6 — Additional file 5: Table S1. Primer sequences for Sanger sequencing. Table S2. Primer sequences for plasmid construction. Table S3. Primer sequences for plasmid construction. Table S4. Dilution of antibody. Table S5. Primer sequences for RT-qPCR. Table S6. Primer sequences for Genotyping. [file 12964_2023_1218_MOESM5_ESM.docx]

| **Table S1. Primer sequences for Sanger sequencing** | |
| --- | --- |
| Primer Name | Primer sequence (5’-3’) |
| ANLN-F | GGGAGAGACCTTGATGATGCTTAT |
| ANLN-R | AAATGCCACCTGAATGCTTTAGAC |

| **Table S2. Primer sequences for plasmid construction** | |
| --- | --- |
| Primer Name | Primer Sequence (5’-3’) |
| FLAG/ANLN-HindⅢ F1 | CAAGCTGGCTAGTTAAGCTTATGGATCCGTTTACGGAGAAAC |
| E841K-R1 | TGGCTACCATATTTTTAGCTCCTGCTTTTAGTAT |
| E841K-F2 | AAAGCAGGAGCTAAAAATATGGTAGCCACACCATT |
| FLAG/ANLN-XbaⅠ R2 | TAGTCGAAGGGCCCTCTAGAAGGCTTTCCAATAGGTTTGTAG |

| **Table S3. Primer sequences for plasmid construction** | |
| --- | --- |
| Primer Name | Primer Sequence (5’-3’) |
| PLJM1/ANLN-EcoRⅠ F1 | CGAGCTCAAGCTTCGAATTCATGGATCCGTTTACGGAGAAAC |
| PLJM1/ANLN-EcoRⅠ R1 | TGTCTCGAGGTCGAGAATTCAGGCTTTCCAATAGGTTTGTAG |

| **Table S4. Dilution of antibody** | |
| --- | --- |
| Antibody | Brand (Proportion of dilution) |
| ANLN | Bethyl (1:1000) |
| GFP | Yeasen (1:5000) |
| Flag | Sigma (1:5000) |
| GAPDH | Proteintech (1:10000) |
| CD2AP | Santacruz (1:1000) |
| mTOR | CST (1:1000) |
| Phospho-mTOR (Ser2448) | CST (1:1000) |
| Bcl-2 | Abclonal (1:5000) |
| Bax2 | Proteintech (1:5000) |
| Cleaved-Caspase3 | CST (1:1000) |
| Caspase3 | Solarbio (1:1000) |
| Nephrin | Abcam (1:5000) |
| AKT | Beyotime (1:1000) |
| Phospho-AKT (Ser473) | Beyotime (1:1000) |
| PI3K | Beyotime (1:1000) |
| Phospho-PI3K (y467/y464/y199) | Beyotime (1:1000) |
| Cyclin E2 | CST (1:1000) |
| p21 | CST (1:1000) |
| p27 | CST (1:1000) |
| DDIT3/CHOP | Abclonal (1:1000) |
| Rac1 | CST (1:1000) |

| **Table S5. Primer sequences for RT-qPCR** | |
| --- | --- |
| Primer Name | Primer Sequence (5’-3’) |
| AKT-F | GTCATCGAACGCACCTTCCAT |
| AKT-R | AGCTTCAGGTACTCAAACTCGT |
| Caspase3-F | GAAATTGTGGAATTGATGCGTGA |
| Caspase3-R | CTACAACGATCCCCTCTGAAAAA |
| mTOR-F | GCAGATTTGCCAACTATCTTCGG |
| mTOR -R | CAGCGGTAAAAGTGTCCCCTG |
| PDK1-F | GAGAGCCACTATGGAACACCA |
| PDK1-R | GGAGGTCTCAACACGAGGT |
| Rac1-F | ATGTCCGTGCAAAGTGGTATC |
| Rac1-R | CTCGGATCGCTTCGTCAAACA |
| IL-6 F | CACTTCACAAGTCGGAGGCT |
| IL-6 R | TCTGACAGTGCATCATCGCT |
| IL-1β F | TAGCAGCTTTCGACAGTGAGG |
| IL-1β R | CTCCACGGGCAAGACATAGG |
| TNF-α F | ATGGGCTCCCTCTCATCAGT |
| TNF-α R | GCTTGGTGGTTTGCTACGAC |
| Actin-F | CATGTACGTTGCTATCCAGGC |
| Actin-R | CTCCTTAATGTCACGCACGAT |

| **Table S6. Primer sequences for Genotyping** | |
| --- | --- |
| Primer Name | Primer Sequence (5’-3’) |
| ANLN mouse-F2 | AGTCTAAGAAAGCGAGTGCAGAA |
| ANLN mouse-R2 | CTCATACAAAGGTAATCGGCATCA |
| ANLN mouse-F1 | GATGTAGACTAGCTGAGTGGATGAG |
| ANLN mouse-R1 | GAAACGACTATGTGCTACGTGTG |
| NPHS2Cre mouse-F | GCGCTGCTGCTCCAG |
| NPHS2Cre mouse-R | CGGTTATTCAACTTGCACCA |
